# Circular RNA circ_UBAP2 facilitates the progression of osteosarcoma by regulating microRNA miR-637/high-mobility group box (HMGB) 2 axis

**DOI:** 10.1080/21655979.2022.2033447

**Published:** 2022-02-03

**Authors:** Weiguo Ma, Xin Zhao, Yun Gao, Xiaobin Yao, Junhua Zhang, Qingxia Xu

**Affiliations:** a Department of Clinical Laboratory, Affiliated Cancer Hospital of Zhengzhou University, Henan Cancer Hospital, Zhengzhou, China; b Zhengzhou Key Laboratory of Digestive Tumor Markers, Cancer Hospital of Zhengzhou University, Zhengzhou China; c Department of Clinical Laboratory, The First Affiliated Hospital of Zhengzhou University, Zhengzhou, China

**Keywords:** Osteosarcoma, circ_UBAP2, miR-637, HMGB2, ceRNA

## Abstract

Circular RNA circ_UBAP2 has been reported to be closely associated with various tumors. The present work focused on exploring the roles of circ_UBAP2 and its molecular mechanism in osteosarcoma (OS). Circ_UBAP2, miR-637, and high-mobility group box (HMGB) 2 levels in OS cells and tissues were detected by quantitative real-time polymerase chain reaction. The relationship between miR-637 and circ_UBAP2, as well as between miR-637 and HMGB2, was predicted and examined through bioinformatics analysis and luciferase reporter gene experiments. Moreover, OS cell growth, invasion, migration, and apoptosis were detected using the cell counting kit-8 (CCK-8), Transwell and flow cytometry assays, respectively. HMGB2 protein levels were measured using Western blotting. Xenograft tumor formation assay was also performed. Circ_UBAP2 showed high expression levels in OS tissues and cells, which was directly proportional to metastasis and clinical stage of OS. The overexpression of circ_UBAP2 enhanced the growth, invasion, and migration of OS cells, but suppressed their apoptosis. In contrast, circ_UBAP2 silencing had opposite effects. Furthermore, miR-637 served as a downstream target of circ_UBAP2, which played opposite roles to circ_UBAP2 in OS. More importantly, HMGB2 served as miR-637ʹs downstream target. The xenograft experiments in nude mice also proved that knockdown of circ_UBAP2 could increase miR-637 expression, but decrease HMGB2 expression, thus alleviating OS progression. Mechanistically, circ_UBAP2 exerts a cancer-promoting effect on OS by downregulating miR-637 and upregulating the expression of HMGB2. Circ_UBAP2 plays a promoting role in OS, and the circ_UBAP2/miR-637/HMGB2 axis is involved in OS progression.

## Introduction

Osteosarcoma (OS) is a common bone cancer. It has a young age of onset and is of great harm [1]. In the early stage, it usually metastasizes to the lungs and has a higher mortality rate [2]. For patients with OS, the current treatment method is still based on surgical resection of the lesion, combined with preoperative and postoperative adjuvant treatments (including adjuvant chemotherapy, immunotherapy, targeted therapy, etc.), and increases patient survival. However, the treatment effect on OS cases with recurrent or metastatic disease is still not optimistic [3]. As a result, OS cases still have a poor survival rate at 5 years, which has become a major disease that seriously threatens people’s health. In this regard, there is an urgent need to identify new and efficient diagnostic/prognostic biomarkers and therapeutic targets for OS patients to develop new treatment strategies to further improve OS survival.

Circular RNAs (circRNAs) are new non-coding RNAs (ncRNAs) that are extensively present in eukaryotic organisms [4]. It is highly conserved in evolution, and its ring structure is stable and difficult to decompose. Research has found that circRNA has a variety of potential biological functions, including miRNA sponge, transcription factor, and gene expression regulation functions, and is an important regulatory factor for tumor occurrence and development [5–7]. It has been reported that circRNAs are closely associated with a variety of tumors, including colon cancer [8], ovarian cancer [9], gastric cancer [10], esophageal cancer (EC) [11], OS [12], and glioma [13]. In OS, circ_0001721 functions as a tumor promoter to facilitate malignant tumor behavior by targeting the miR-372-3p/mitogen-activated protein kinase 7 axis (MAPK7) [14]. Circ_0003074, screened by chip sequencing, showed high expression in OS tissues, which can be recognized as a marker for the diagnosis and treatment of OS [15]. Circ_UBAP2 has been reported to promote triple-negative breast cancer (BC), glioma, and prostate cancer progression [16–18]. However, the role of circ_UBAP2 in OS and the possible molecular mechanisms remain unclear.

miRNAs are single-stranded ncRNAs that contain approximately 22 nucleotide sequences in length. They are the key molecules that can regulate gene expression and widely exist in organisms and are closely related to the growth and development, physiology and pathology, aging, and death processes of organisms [19,20]. miRNAs are also involved in the development of OS and regulate cell proliferation, migration, and apoptosis [21]. For example, miR-17-5p targets Src kinase signaling inhibitor 1 (SRCIN1) to facilitate OS cell growth and epithelial-mesenchymal transition [22]. MiR-183 impedes OS malignant phenotypes by targeting metastasis-associated protein 1 (MTA1) [23]. MiR-637 functions as a tumor suppressor gene in melanoma, cholangiocarcinoma, and hepatoma cells [24–26]. Despite this, the expression level of miR-637 in OS and the underlying mechanism remain largely unclear.

circRNAs are widely suggested to sponge miRNAs and function as competing endogenous RNAs (ceRNAs), which then interfere with the binding of miRNAs to target gene sequences, thus affecting the regulation of gene expression [27]. As discovered by Wu et al., the expression of circTADA2A increased in OS tissues, which sponged miR-203a-3p as the ceRNA to regulate cyclic AMP-responsive element-binding protein 3 (CREB3) level, thereby affecting OS malignant tumor behavior [28]. Ji et al. stated that circ_001621 enhanced OS cell proliferation and invasion by sponging miR-578, which subsequently regulates the vascular endothelial growth factor (VEGF) level [29]. Circ_UBAP2 has been reported to function as a sponge of miR-1205, miR-382, miR-199a, miR-1244, and miR-204-3p to promote the progression of several tumors [17,18,30,31]. Nevertheless, whether circ_UBAP2 sponges miR-637 to regulate OS malignant behavior has not been studied.

HMGB2, a member of the HMG protein family, is an important protein in carcinogenesis. In recent years, HMGB2 has been shown to be overexpressed in various cancers, including pancreatic cancer, melanoma, and BC [32–34]. In addition, HMGB2 overexpression predicts the survival of patients with primary hepatocellular carcinoma [35]. More importantly, HMGB2 was suggested to promote the progression of OS and may serve as a new target for OS research [36]. Studies have shown that silencing HMGB2 inhibits BC cell growth, migration, and invasion. HMGB2 is reported to be a target of miR-329, miR-590-3p, miR-23b-3p, and miR-130a [33,37–39]. However, we do not know whether HMGB2 is a possible miR-637 target in OS.

The present study aimed to investigate the function of circ_UBAP2 and its underlying mechanisms in OS. The present study confirmed for the first time that the circ_UBAP2/miR-637/HMGB2 axis participates in OS progression, which offers a novel approach for OS treatment. We hypothesized that circ_UBAP2 affects OS cell proliferation, migration, and invasion via the miR-637/HMGB2 axis.

## Materials and methods

### Clinical specimens

This study obtained 40 human OS specimens from OS patients undergoing surgical resection for OS at the Affiliated Cancer Hospital of Zhengzhou University. After collection, the samples were preserved in liquid nitrogen. Each patient provided written consent for participation. The Ethics Committee of Zhengzhou University approved the study protocol. The inclusion criteria were as follows: i) tissues were obtained during surgery and diagnosed with OS by two pathologists; ii) patients who did not receive chemotherapy or radiotherapy; and iii) patients who are willing to participate. The exclusion criteria were as follows: i) patients with other diseases, including other tumors; ii) patients who received treatment prior to participation in the present study; and iii) patients who refused to participate in the study.

### Cell lines and transfection

OS cells (U-2OS, MG63, SaOS-2, and HOS) and healthy osteoblasts (hFOB1.19) were provided by the Chinese Academy of Sciences (Shanghai, China) and cultivated in Dulbecco’s modified eagle’s medium containing 10% fetal bovine serum (Gibco, Thermo Fisher Scientific, Inc.). hFOB1.19 cells were cultured in a humidified incubator at 33.5°C, and OS cells were cultured at 37°C [40].

Small interfering RNAs (si-RNAs), miR-637 mimic (or mimic NC), and miR-637 inhibitor (inhibitor NC) were obtained from GenePharma. In line with specific protocols, HOS and SaOS-2 cells were cultivated in 6-well plates, followed by 48 h of transfection using si-RNAs or miR-637 mimic or inhibitor with Lipofectamine^3000^ (Invitrogen) [40]. Next, we calculated the transfection efficiency.

### Quantitative reverse transcriptase-PCR (qRT-PCR)

Trizol reagent (Thermo Fisher Scientific, Waltham, MA, USA) was used to extract total cellular and tissue RNA. The purity and content of extracted total RNA were then measured. Later, the Prime Script Rt Reagent Kit (TaKaRa, Dalian, China) was used to synthesize cDNA through reverse transcription, whereas qRT-PCR was performed using SYBR Premix Ex Taq (TaKaRa, Dalian, China), with GAPDH and U6 as endogenous references. The 2^−ΔΔCT^ approach was used to quantify the relative levels of circ_UBAP2, HMGB2, and miR-637. Table 1 lists primer sequences [41].

**Table 1.** Sequences used for qRT-PCR

Gene		Sequence (5`to3`)
circUBAP2 primer miR-637 primer HMGB2 primer U6 primer GAPDH primer	F R F R F R F R F R	CCAGTTCTTAGCCAGTTGA TGTCTCCAGGTGTTGATTC AGCCCACACACTACAGGCA GCACAAAAGCAGTACGACCT TATGGCAAAAGCGGACAAGG CTTCGCAACATCACCAATGGA CTCGCTTCGGCAGCACATATACT ACGCTTCACGAATTTGCGTGTC CTCCATCCTGGCCTCGCTGT GCTGTCACCTTCACCGTTCC

### Cell counting kit-8 (CCK-8) assay

After transfection, the HOS and SaOS-2 cells were cultured in 96-well plates. Fresh complete culture medium (100 μL) was replaced at an interval of 24 h (90 μL fresh complete culture and 10 μL CCK-8 solution into all wells), followed by incubation in the incubator for another 1 h. A microplate analyzer was used to determine absorbance at 450 nm (A450) [42].

### Transwell assay

After 48 h of transfection, trypsin was used to digest the cells from all groups. Afterward, cells were harvested and resuspended in relevant serum-free medium at 2 × 10^4^ cells/mL. To measure cell migration, we added a certain volume of cell suspension (200 μL) into the top Transwell chamber, whereas complete medium was added to the lower chamber. Following culture for 24 h under 5% CO_2_ and 37°C conditions, the medium was removed, and cotton swabs were utilized for wiping on the top chamber surface. After rinsing with PBS, the cells were subjected to 30 min of 4% paraformaldehyde fixation, 0.1% crystal violet staining for 10 min, observation under a microscope, and photographed in five random fields. To measure cell invasion, we used Transwell chambers pre-coated with Matrigel (pore size, 8 μM) (Corning, Beijing, China). Matrigel was diluted in Opti-MEM culture medium at 1:8, followed by the addition of 50 μL) in the top Transwell chamber. After drying at room temperature, cell migration experiments were performed. Finally, the number of cells stained with crystal violet was calculated microscopically as the number of invasive cells [43].

### Subcellular fractionation assay

ReadiPrep nuclear/cytoplasmic fractionation kit (AAT Bioquest, USA) was used to value the location of circ_UBAP2. Briefly, cells were treated with cytosol extraction buffer, centrifuged and the supernatant was collected as a cytoplasmic extract. The precipitates were suspended and collected as a nuclear extract after centrifugating. Finally, RT-qPCR assay was to detect circ_UBAP2 expression in the nuclear and cytoplasmic fractions. Internal cytoplasmic reference was GAPDH, U6 represented the nuclear RNA control [44].

Biotin pull-down assay

Biotin-labeled wild-type miR-637 (Bio-wt-miR-637), mutant miR-580-3p (Bio-mut-miR-637) or negative control (NC) were transfected into HOS and SaOS-2 cells. After transfection for 48 h, cells were lysed using lysis buffer. Then cell lysate was incubated with M-280 streptavidin magnetic beads (Invitrogen, USA) and RT-PCR was used to determin bound RNA expressions [44].

### Western blotting (WB) assay

Cells transfected for 48 h were harvested from all groups, followed by lysis using RIPA lysis buffer (Beyotime, Shanghai, China). The cells were then centrifuged for 15 min at 12,000 g and 4°C to collect protein supernatants, and the BCA kit (Thermo Fisher Scientific, Inc.) was used to measure protein content. Later, proteins were separated through sodium dodecyl sulfate-polyacrylamide gel electrophoresis, and then transferred onto polyvinylidene fluoride (PVDF) membranes (Millipore, Bedford, MA, USA) as well as 2 h of blocking using 5% nonfat milk at ambient temperature. The primary antibodies (Abcam, Shanghai, China), anti-HMGB2 (ab124670, 1: 10,000), and anti-GAPDH (ab9485, 1:2500) were added, followed by an overnight incubation at 4°C. The next day, the membrane was rinsed with Tris-buffered saline with Tween-20 (TBST) and incubated with horseradish peroxidase (HRP)-labeled secondary antibody (ab205718, 1:5000) for 2 h at 37°C. After the PVDF membranes were washed with TBST three times, bands were visualized using ECL chemiluminescence (Beyotime, Shanghai, China) [40].

### Flow cytometry analysis

Apoptosis was detected using an Annexin V-FITC/PI apoptosis assay kit. At 48 h after transfection, HOS and SaOS-2 cells were digested with 0.25% trypsin and re-suspended twice with binding buffer. Thereafter, every tube was added with Annexin V-FITC (5 µL) and propidium iodide (PI, 10 μL) for 10 min, followed by incubation at ambient temperature. Finally, cell apoptosis was detected by flow cytometry after 1 h [43].

### Luciferase reporter assay

Circinteractome and TargetScan databases, respectively, indicated that circUBAP2 and HMGB2 3 ‘UTR regions had miR-637 binding sites. In this study, we constructed wild-type (WT) circUBAP2 (circUBAP2-wt), HMGB2 3’ UTR (HMGB2-wt), and mutant circUBAP2 (circUBAP2-mut) and HMGB2 (HMGB2-mut) luciferase reporter vectors. Thereafter, we co-transfected these vectors with mimic NC or miR-637 mimic into HOS and SaOS-2 cells. At 48 h later, we used the Dual-Luciferase Reporter Assay System (Promega, WI, USA) to quantify luciferase activity [43].

### Xenograft tumor formation

The male nude mice (four to six-week, weighing approximately 26 g) were purchased from the Animal Experimental Center of Henan Province (Zhengzhou, China). All animal experiments and programs were approved by Zhengzhou University. HOS cells (3 × 10^6^) with sh-circ_UBAP2 or sh-NC were injected into mice. After 5 weeks, the mice were sacrificed, and the tumors were weighed. Tumor size was calculated using the following equation: tumor volume [mm^3^] = (length [mm]) × (width [mm])^2^ × 0.52 [45].

### Statistical analysis

SPSS20.00 was used for Statistical analysis. We presented the measurement data in the form of mean ± standard deviation. Comparisons between two groups were conducted using the t-test, while those across several groups were analyzed using one-way analysis of variance. Pearson’s correlation analysis was used to analyze the associations between gene expression levels. Statistical significance was set at *P* < 0.05.

## Results

In this study, we explored the role of circ_UBAP2 in OS. A series of assays demonstrated that circ_UBAP2 promotes OS progression by regulating the miR-637/HMGB2 axis. Our findings highlight the functional roles of circ_UBAP2 in OS, providing new insights into OS pathogenesis.

### Circ_UBAP2 is highly expressed in OS and its clinical significance

**Figure 1.**
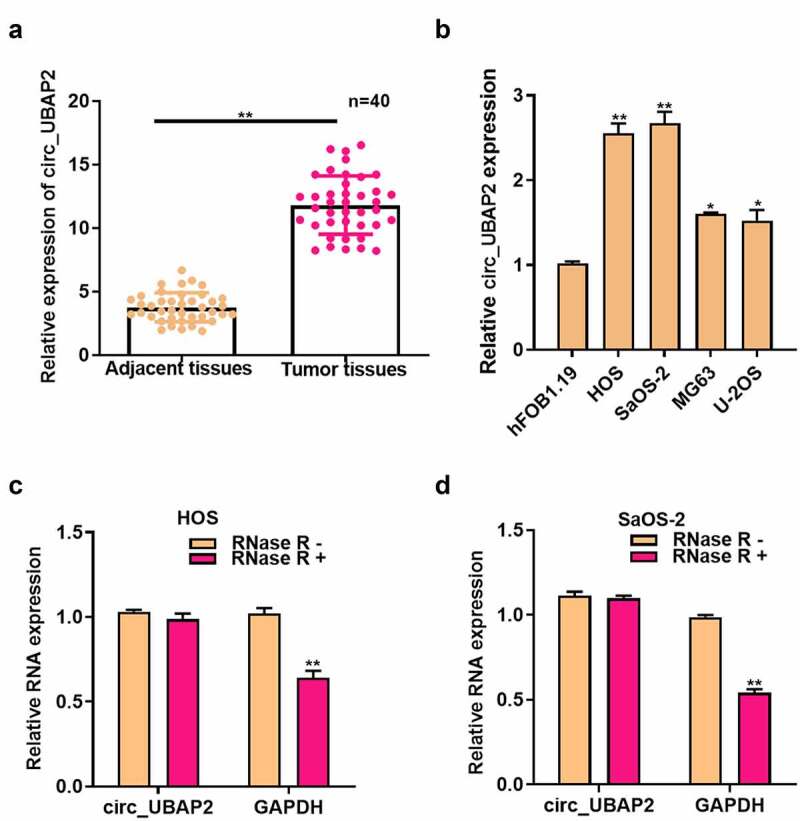
Circ_UBAP2 is highly expressed in OS.a). circ_UBAP2 levels within OS and non-carcinoma tissues (n = 40) were measured by qRT-PCR. b). circ_UBAP2 levels within OS cells (U-2OS, MG63, SaOS-2, HOS) and healthy osteoblasts (hFOB1.19) were detected by qRT-PCR. c). RNase treated in HOS cells, circ_UBAP2 and GAPDH expression was measured to confirm the higher stability of circ_UBAP2 than the linear RNA. d). RNase treated in SaOS-2 cells, circ_UBAP2 and GAPDH expression was measured to confirm the higher stability of circ_UBAP2 than the linear RNA. *P < 0.05, ** *P* < 0.01.

First, circ_UBAP2 levels in OS tissues were measured using qRT-PCR and showed a significantly increased levels in OS tissues (Figure 1(a)). In addition, we demonstrated that circUBAP2 levels were markedly related to distant metastasis and tumor, node, metastasis stage (Table 2). qRT-PCR results showed that compared with human osteoblast hFOB1.19, circ_UBAP2 expression in the four OS cells was markedly elevated, particularly in HOS and SaOS-2 cells (Figure 1(b)). In addition, after RNase R treatment, GAPDH and circ_UBAP2 expression was measured in HOS and SaOS-2 cells. It was discovered that circ_UBAP2, rather than GAPDH, was resistant to RNase R, which implied the circRNA characteristics of circUBAP2 (Figure 1(c,d)). These data suggest that high circ_UBAP2 expression is related to OS progression.

**Table 2.** Correlations of circUBAP2 with clinical characteristics in osteosarcoma patients

Clinical parameters	Cases	circUBAP2 expression		P value
		Low (n = 20)	High (n = 20)	
Gender				0.751
Male	22	10	12	
Female	18	10	8	
Age (years)				0.748
<18	24	13	11	
≥18	16	7	9	
Location				0.343
Femur 20 12 8				
Other 20 8 12				
Tumor size (cm)				0.461
<8	22	15	7	
≥8	18	5	13	
TNM stage				0.048*
I–II	15	11	4	
III	25	9	16	
Distant metastasis				0.010*
No	19	14	5	
Yes	21	6	15	

*P < 0.05.

### The roles of circ_UBAP2 on OS cell growth, invasion, migration, and apoptosis

**Figure 2.**
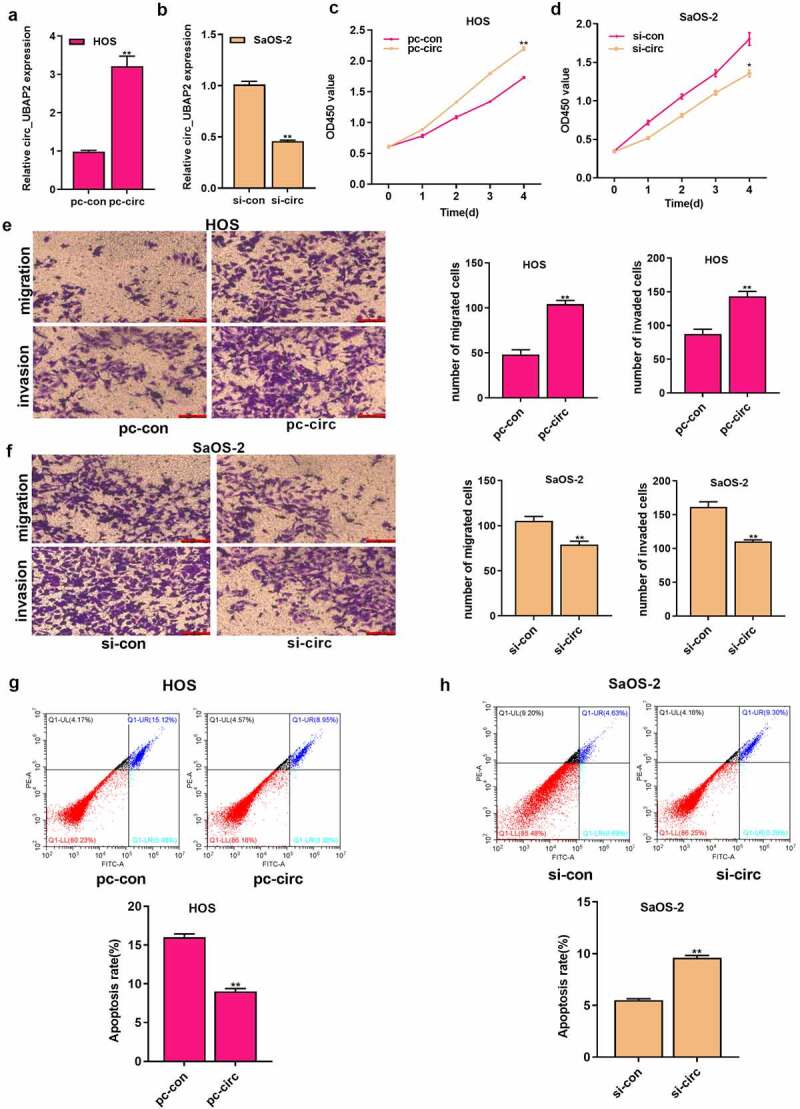
Roles of circ_UBAP2 on proliferation, migration, invasion and apoptosis of OS cells.a) circ_UBAP2 level was measured following the transfection of pc-circ_UBAP2 (or pc-control) into HOS cells through qRT-PCR. b) circ_UBAP2 levels were measured following the transfection of circ_UBAP2 siRNA (or siRNA NC) in SaOS-2 cells via qRT-PCR. c) Transfected HOS cell proliferation was measured through CCK-8 assay. d) Transfected SaOS-2 cell proliferation was measured through CCK-8 assay. e) Invasion and migration of transfected HOS cells analyzed by Transwell assay. f) Invasion and migration of SaOS-2 cells after the transfection analyzed by Transwell assay. g) Transfected HOS cell apoptosis measured through flow cytometry. h) Transfected SaOS-2 cell apoptosis detected through flow cytometry. * *P* < 0.05, ** *P* < 0.01.

Circ_UBAP2 upregulation was detected, particularly in HOS and SaOS-2 cells, which were chosen for subsequent cell function assays. pc-circ_UBAP2 (or pc-NC) was transfected into HOS cells, whereas circ_UBAP2 siRNA (or siRNA NC) in SaOS-2 cells to construct cell models with overexpression (gain-of-function) and knockdown (loss-of-function). As revealed by qRT-PCR, circ_UBAP2 was upregulated in HOS cells with circ_UBAP2 overexpression, whereas it was downregulated in SaOS-2 cells with circ_UBAP2 silencing (Figure 2(a,b)), which demonstrated that the transfection was successful. We then investigated how circ_UBAP2 affected OS cell growth, invasion, migration, and apoptosis. As found from diverse assays, overexpression of circ_UBAP2 remarkably enhanced the growth, invasion, and migration of HOS cells, but inhibited their apoptosis. However, it was demonstrated that circ_UBAP2 knockdown significantly blocked SaOS-2 cell growth, invasion, and migration, and enhanced apoptosis (Figure 2(c-h)). These data showed that circ_UBAP2 acts as an oncogene in OS.

### MiR-637 is the target of circ_UBAP2

**Figure 3.**
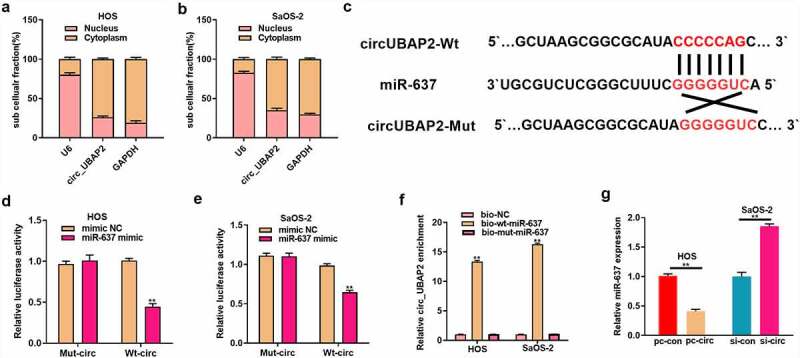
MiR-637 is the circ_UBAP2 target.a) qRT-PCR detected subcellular localization of circ_UBAP2 in HOS cells. b) qRT-PCR detected subcellular localization of circ_UBAP2 in SaOS-2 cells. c) Binding sequences of miR-637 with circ_UBAP2 predicted with bioinformatics. d) Targeting relation of miR-637 with circ_UBAP2 was measured through dual-luciferase reporter assay within HOS cells. e) Targeting relation of miR-637 with circ_UBAP2 was measured through dual-luciferase reporter assay within SaOS-2 cells. f) qRT-PCR detected expression of streptavidin-captured circ_UBAP2 after biotin-labeled miR-637 (bio-wt-miR-637), bio-mut-miR-637 or negative control (bio-NC) were transfected into OS cells. g) miR-637 levels within OS cells were detected through qRT-PCR upon circ_UBAP2 over-expression within HOS cells or silencing within SaOS-2 cells. * *P* < 0.05, ** *P* < 0.01.

CircRNA modulates gene levels by combining with miRNAs and blocks the inhibition of miRNA target genes, thus participating in the mechanism of tumorigenesis [7,8]. To investigate the regulatory mechanism of circ_UBAP2, intracellular location of circ_UBAP2 was determined. Subcellular fractionation assay indicated that circ_UBAP2 was predominantly localized in the cytoplasm (Figure 3(a,b)). Then we sought to identify the miRNAs that bind to circ_UBAP2 through bioinformatics analysis, and found that miR-637 was one of them (Figure 3(c)). Subsequently, we conducted a dual-luciferase reporter assay, which revealed that the WT reporter gene of the miR-637 mimic significantly inhibited the luciferase activity of the circ_UBAP2 WT reporter, which suggested that miR-637 bound to circ_UBAP2 within OS (Figure 3(d,e)). RNA pull-down assay also validated the interplay between circ_UBAP2 and miR-637 (Figure 3(f)). Moreover, miR-637 levels were downregulated following the overexpression of circ_UBAP2 in HOS cells, but increased after circ_UBAP2 knockdown in SaOS-2 cells (Figure 3(g)). Collectively, these results verified that circ_UBAP2 targeted miR-637 and showed a negative regulation of its level within OS.

### MiR-637 plays a tumor-suppressive role in OS

**Figure 4.**
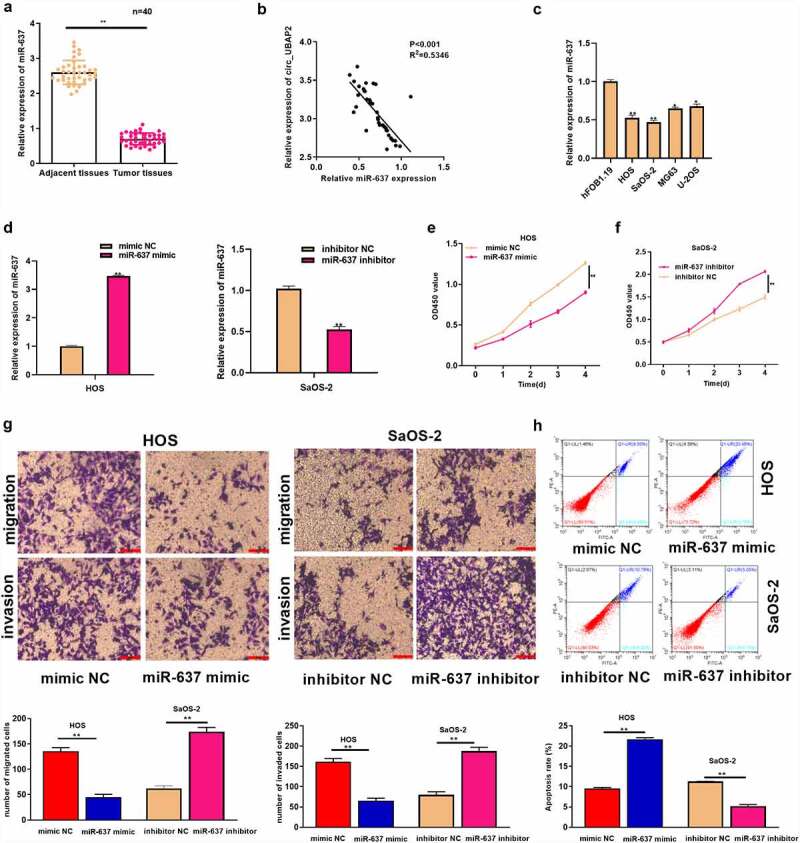
MiR-637 functions a tumor suppressive effect on OS.a) miR-637 levels were measured within OS and corresponding non-carcinoma tissues (n = 40) by qRT-PCR. b) miR-637 level showed negative correlation with circ_UBAP2 levels within OS tissues. c) miR-637 levels within OS cells (U-2OS, MG63, SaOS-2, HOS) and healthy osteoblasts (hFOB1.19) were measured by qRT-PCR. D) miR-637 levels were measured upon co-transfection of miR-637 inhibitor (or inhibitor NC) with miR-637 mimic (or mimic NC) in OS cells through qRT-PCR. e) Ttransfected HOS cell proliferation was detected through CCK-8 assay. f) Transfected SaOS-2 cell proliferation was detected through CCK-8 assay. g) Invasion and migration of transfected HOS and SaOS-2 cells were analyzed through Transwell assays. h) Transfected HOS and SaOS-2 cell apoptosis was analyzed through flow cytometry. *P < 0.05, ** *P* < 0.01.

MiR-637 has been suggested to participate in cancer development [24–26]. However, its role in OS has not been fully elucidated. This study adopted qRT-PCR assay which was used to detect miR-637 levels within OS tissues and showed a low expression (Figure 4(a)), which was in indirect proportion to circ_UBAP2 levels within OS tissues (Figure 4(b)). miR-637 levels were remarkably decreased in OS cells (Figure 4(c)). We transfected miR-637 mimic (or mimic NC) in HOS cells, whereas miR-637 inhibitor (or inhibitor NC) was transfected into SaOS-2 cells to explore miR-637ʹs function in OS. miR-637 levels were elevated in HOS cells with miR-637 overexpression and declined within SaOS-2 cells with miR-637 silencing, as revealed by qRT-PCR (Figure 4(d)), which demonstrated that the transfection was successful. We then explored miR-637ʹs function in OS cell growth, invasion, migration, and apoptosis. Through a series of experiments, it was observed that miR-637 overexpression significantly inhibited HOS cell growth, invasion, and migration, but enhanced apoptosis. In contrast, decreased miR-637 expression remarkably enhanced SaOS-2 cell growth, invasion, and migration, but damaged cell apoptosis (Figure 4(e-h)). These results suggest the anticancer activity of miR-637 in OS.

### MiR-637 partially reverses the tumor-promoting effect of circ_UBAP2 on OS cells

**Figure 5.**
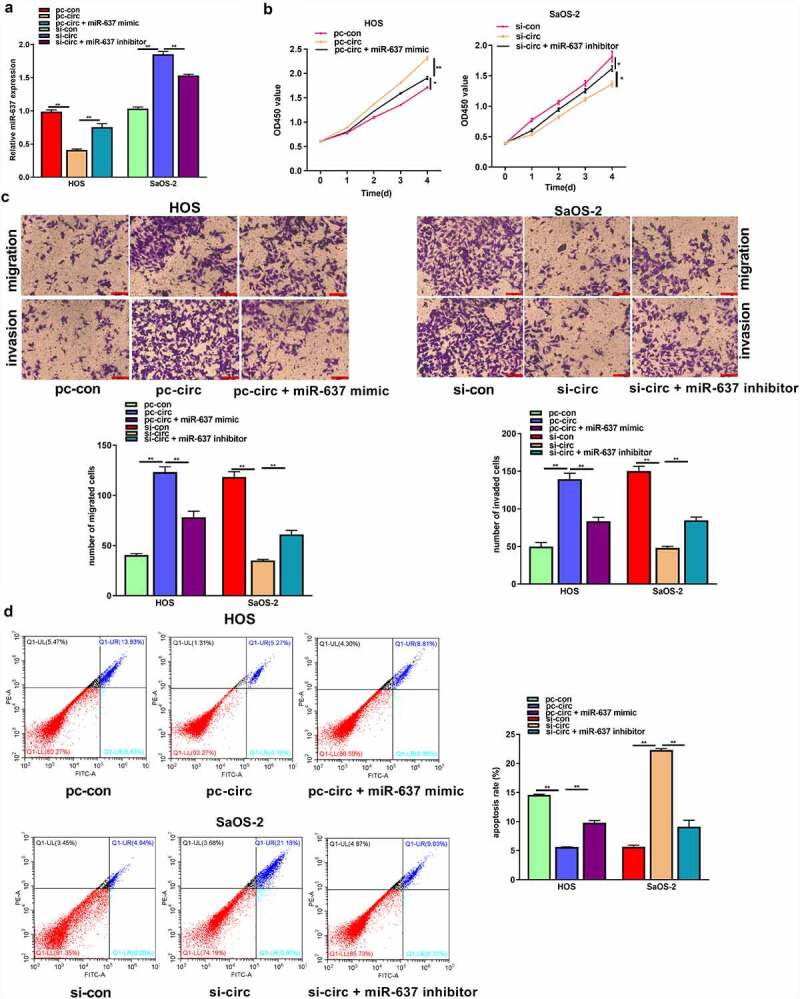
MiR-637 partially reverses the tumor-promoting effect of circ_UBAP2 on OS cells.HOS cells with stable over-expression of circ_UBAP2 were transfected with miR-637 mimic, while SaOS-2 cells with circ_UBAP2 silencing were subjected to miR-637 inhibitor transfection. a) miR-637 expression within OS cells was analyzed through qRT-PCR. b) Transfected HOS and SaOS-2 cell proliferation was detected through CCK-8 assay. c) Invasion and migration of transfected HOS and SaOS-2 cells were analyzed through Transwell assays. d) Transfected HOS and SaOS-2 cell apoptosis was analyzed through flow cytometry. *P < 0.05, **P < 0.01.

To explore the effect of the circ_UBAP2/miR-637 axis on OS, we transfected circ_UBAP2-overexpressed HOS cells with miR-637 mimic, whereas circ_UBAP2-silencing SaOS-2 cells were transfected with the miR-637 inhibitor. According to the qRT-PCR assay, miR-637 levels declined within circ_UBAP2-overexpressed HOS cells, but partially increased when cells were transfected with the miR-637 mimic. In SaOS-2 cells, miR-637 expression was increased in the circ_UBAP2 knockdown SaOS-2 cells, but partially decreased when cells were transfected with an miR-637 inhibitor, demonstrating successful transfection (Figure 5(a)). Through a series of functional experiments, miR-637 overexpression was found to offset the impact of circ_UBAP2 upregulation on HOS cell growth, invasion, and migration, but suppressed their apoptosis. miR-637 downregulation partially reversed the inhibitory effects of circ_UBAP2 knockdown on SaOS-2 cell growth, invasion, and migration, but promoted apoptosis (Figure 5(b-d)). Based on the above findings, the circ_UBAP2/miR-637 axis is involved in OS progression.

### HMGB2 is the miR-637 target

**Figure 6.**
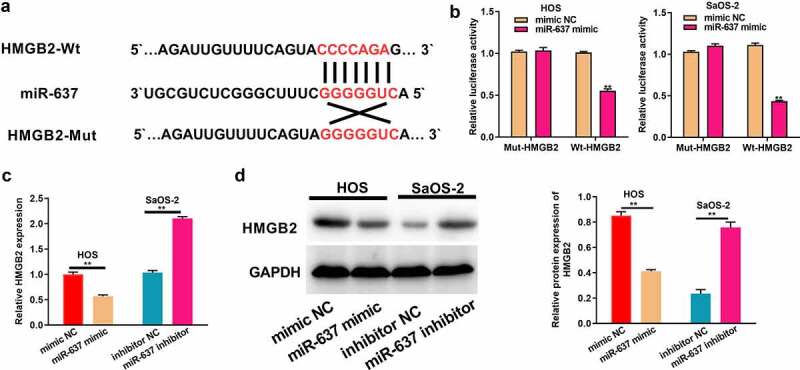
HMGB2 is the target of miR-637.a) Bioinformatics analysis conducted for predicting binding relation of miR-637 with HMGB2. b) The targeting relation of miR-637 with HMGB2 within OS cells was confirmed through dual-luciferase reporter assay. c) HMGB2 levels within OS cells following miR-637 mimic transfection in HOS cells or miR-637 inhibitor transfection into SaOS-2 cells were analyzed through qRT-PCR. d) HMGB2 expression within OS cells following miR-637 mimic transfection in HOS cells or miR-637 inhibitor transfection into SaOS-2 cells was analyzed through WB assay. *P < 0.05, **P < 0.01.

MiRNAs can recognize the 3’-UTR of target mRNAs to achieve negative regulation of target gene expression by suppressing target mRNA translation or decreasing its stability at the post-transcriptional level [19–21]. Therefore, the downstream genes of miR-637 were identified, and it was found that HMGB2 was one of them (Figure 6(a)). As revealed by dual-luciferase reporter assays, the WT reporter gene of miR-637 mimic significantly inhibited HMGB2 WT reporter luciferase activity, rather than that of the mutant reporter, which suggested that miR-637 was bound to HMGB2 within OS (Figure 6(b)). It was also observed that HMGB2 mRNA and protein expression levels declined after miR-637 was overexpressed in HOS cells, but increased after miR-637 was knocked down in SaOS-2 cells (Figure 6(c,d)). All these data proved that HMGB2 is the target of miR-637 in OS.

### Circ_UBAP2 promotes OS progression by regulating HMGB2

**Figure 7.**
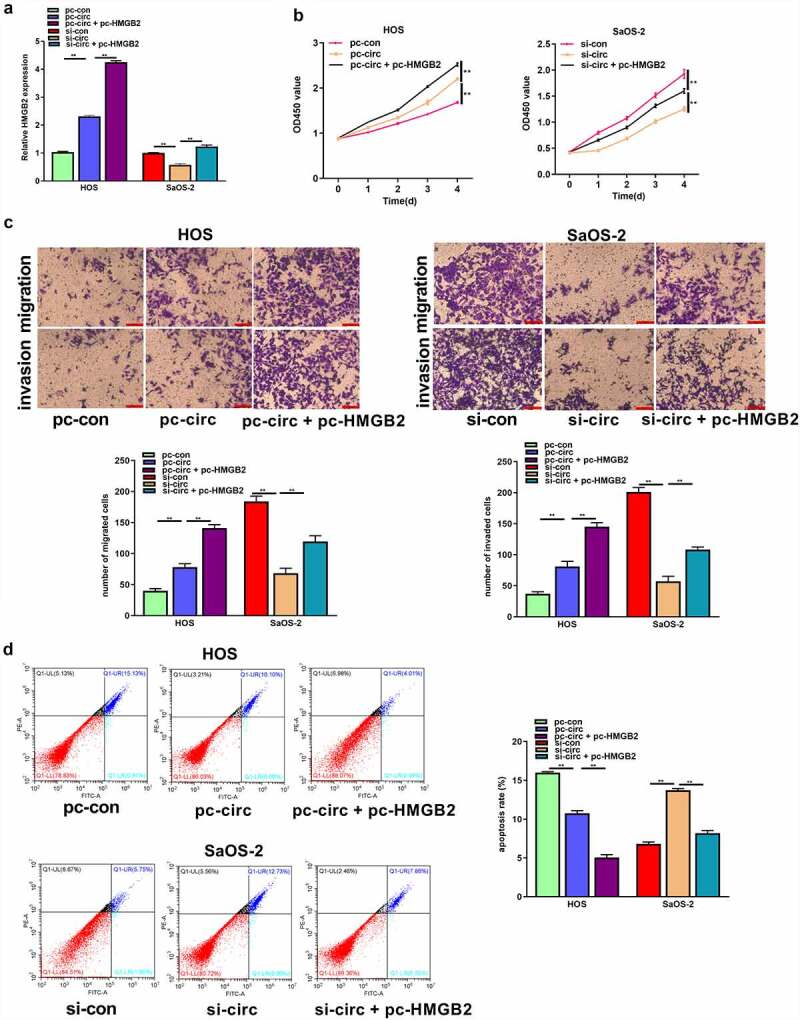
Circ_UBAP2 promotes OS progression by regulating HMGB2.HOS cells with stable over-expression of circ_UBAP2 and SaOS-2 cells with circ_UBAP2 silencing were subjected to pc-HMGB2 transfection, respectively. a) HMGB2 levels within OS cells were analyzed through qRT-PCR. b) Transfected HOS and SaOS-2 cell proliferation was evaluated through CCK-8 assay. c) Transfected HOS and SaOS-2 cell invasion and migration were measured through Transwell assays. d) Transfected HOS and SaOS-2 cell apoptosis was analyzed through flow cytometry. * *P* < 0.05, **P < 0.01

Based on these observations, circ_UBAP2 enhanced the malignant behavior of OS cells, and HMGB2 was a target of miR-637. Therefore, we determined whether circ_UBAP2 promoted the malignant phenotype of OS cells by regulating HMGB2 expression. pc-HMGB2 was transfected into HOS cells with circ_UBAP2 overexpression or circ_UBAP2 knockdown in SaOS-2 cells. qRT-PCR revealed the upregulation of HMGB2 in HOS cells overexpressing circ_UBAP2, and was even more pronounced when cells were co-transfected with pc-HMGB2. HMGB2 expression was decreased in the circ_UBAP2 knockdown SaOS-2 cells, but was partially reversed when cells were co-transfected with pc-HMGB2 in the circ_UBAP2 knockdown SaOS-2 cells (Figure 7(a)). Finally, by performing a series of functional experiments, we demonstrated that HMGB2 upregulation increased the function of circ_UBAP2 overexpression, which promoted HOS cell proliferation, migration, and invasion, but suppressed their apoptosis. Nonetheless, circUBAP2 knockdown-mediated effects were significantly reversed by HMGB2 overexpression (Figure 7(b-d)). These results indicate that circ_UBAP2 promotes OS progression by regulating HMGB2 expression.

### Circ_UBAP2 promotes OS tumor growth in vivo by regulating miR-637/HMGB2 axis

**Figure 8.**
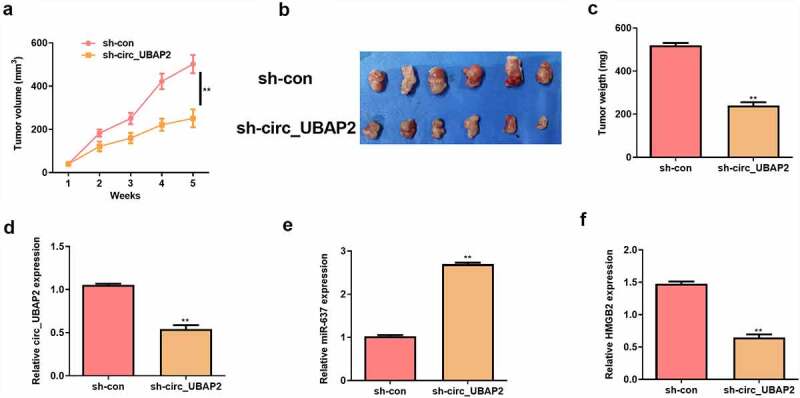
Circ_UBAP2 promotes OS tumor growth in vivo by regulating miR-637/HMGB2 axis.HOS cells stably transfected with sh-circ_UBAP2 or sh-con were subcutaneously injected into mice. a) Tumor volume was calculated. b) Representative pictures of tumor were presented. c) Tumor weight was calculated on the day mice were sacrificed. d) qRT-PCR was to detect the expression of circ_UBAP2. e) qRT-PCR was to detect the expression of miR-637. F) qRT-PCR was to detect the expression of HMGB2.

To investigate the promoting effect of circ_UBAP2 in vivo, a xenografted model was established using HOS cells stably transfected with sh-circ_UBAP2 or sh-con. Five weeks later, tumor volume and weight were reduced in the sh-circ_UBAP2 group (Figure 8(a-c)). Meanwhile, circ_UBAP2 and HMGB2 expression were down-regulated, but miR-637 expression was up-regulated in the sh-circ_UBAP2 group (Figure 8(d-f)). These results verify that circ_UBAP2 promotes tumor growth in vivo by regulating miR-637/HMGB2 axis.

## Discussion

OS is a frequently occurring primary bone cancer among adolescents, and its pathogenic mechanism has been widely explored. With the development of RNA sequencing technology, numerous ncRNAs have been verified to participate in regulating OS [21,28,46,47]. In the field of non-coding RNA, as circRNA has become a research focus, some circRNAs have been verified to participate in OS genesis, and it is a possible marker for the diagnosis and treatment of OS. For example, circ_0000285 is upregulated in OS and promotes OS malignant behavior by the miR-409-3p/insulin-like growth factor-binding protein 3 (IGFBP3) axis [48]. Cheng et al. reported that circVRK1 suppressed the proliferation, migration, and invasion of OS cells by regulating zinc finger protein ZNF652 expression via microRNA miR-337-3p [49]. In addition, Zhang et al. demonstrated that circRNA 0102049 suppressed OS progression by modulating the miR-520 g-3p/PLK2 axis [44]. Circ_0084582 facilitated cell growth, migration, invasion, and angiogenesis in OS by mediating the miR-485-3p/JAG1 axis [50]. As shown in previous studies, circ_UBAP2 upregulation enhances cancer cell growth, invasion, and migration, but suppresses apoptosis, thereby exerting a carcinogenic role during cancer occurrence. As verified in this study, circ_UBAP2 was overexpressed in OS cells and tissues; furthermore, the upregulated expression of circ_UBAP2 enhanced OS cell growth, invasion, and migration, and inhibited their apoptosis. On the contrary, inhibition of circ_UBAP2 had the opposite effect on OS. In conclusion, we demonstrated that circ_UBAP2 promotes multiple malignant biological behaviors of OS at the cellular level. In addition to the cell level, we detected circ_UBAP2 in the clinical tissue samples. As a result, circ_UBAP2 expression was directly proportional to distant metastasis and the clinical stage of OS. These results were consistent with those at the cellular level, suggesting that circ_UBAP2 plays a promoting role in OS.

miRNAs have been extensively suggested to participate in the entire process of tumorigenesis and development, and play a role as tumor suppressors or oncogenes in numerous cellular processes [21–23]. MiR-637, a recently identified miRNA, was found by Zhang and colleagues to be markedly downregulated in colorectal cancer (CRC), while its overexpression suppressed CRC cell growth [51]. Yi et al. showed that miR-637 was under-expressed in glioma, and its abnormal expression might be related to processes such as cancer cell growth and migration [52]. Moreover, miR-637 expression has been found to markedly decrease in ovarian cancer, while its overexpression can inhibit the epithelial mesenchymal transformation process in ovarian cancer [53]. Our results verified the decreased levels of miR-637 in OS cells and tissues. Most importantly, we also demonstrated that upregulation of miR-637 remarkably inhibited OS cell growth, invasion, and migration, but enhanced apoptosis. Moreover, miR-637 downregulation had opposite effects, consistent with prior results suggesting that miR-637 is a tumor suppressor in hepatoma, cholangiocarcinoma, and melanoma.

As verified in numerous articles, the effect on miRNA sponges is the key mechanism by which circRNAs regulate the occurrence and development of diseases [14,16]. For example, Zhang et al. proved that circ_0136666 promotes OS malignant behavior by sponging miR-593-3p and regulating ZEB2 expression [54]. In addition, circHIPK3 promoted OS metastasis by acting on the miR-637/signal transducer and activator of transcription 3 (STAT3) axis [55]. This work confirmed that miR-637 is a downstream target of circ_UBAP2. In addition, miR-637 mimics offset the role of circ_UBAP2 overexpression in OS cells’ malignant behaviors, whereas miR-637 inhibitor reversed the inhibition of circ_UBAP2 silencing on the malignant behavior of OS cells. In conclusion, circ_UBAP2, as a ceRNA, is involved in OS progression by inhibiting miR-637.

HMGB2 is involved in tumorigenesis and progression [56–58]. In this study, HMGB2 was predicted and verified as a possible miR-637 target in OS. Moreover, the overexpression of HMGB2 offset the inhibition of circ_0000144 knockdown on the malignant biological behavior of OS cells, but increased the function of circ_UBAP2 overexpression in enhancing OS cell growth, migration, and invasion, but suppressed their apoptosis, indicating that circ_UBAP2 promoted OS progression by regulating HMGB2. Further tumor xenograft experiments in nude mice also demonstrated that circ_UBAP2 knockdown could increase the expression of miR-637, but decrease the HMGB2 expression, thus promoting OS progression in vivo. Collectively, these data indicate that circ_UBAP2 promotes OS progression by downregulating miR-637 and upregulating HMGB2 expression.

## Conclusion

To sum up, the present work verified the effect of circ_UBAP2 on promoting the growth, invasion and migration of OS cells, and suppressed their apoptosis. Mechanistically, it was first demonstrated that circ_UBAP2/miR-637/HMGB2 axis involved in OS progression. Our study also provides new ideas and targets for the clinical treatment of OS.

## Data Availability

The datasets used and/or analyzed during the current study are available from the corresponding author upon reasonable request.

## Article highlights

- Circ_UBAP2 acted as a sponge of miR-637.
- Circ_UBAP2 promoted HMGB2 expression by suppressed miR-637.
- Circ_UBAP2 promoted osteosarcoma progression through miR-637/HMGB2 axis.
